# Physicochemical and Biological Properties of Polysaccharides from *Dictyophora indusiata* Prepared by Different Extraction Techniques

**DOI:** 10.3390/polym13142357

**Published:** 2021-07-19

**Authors:** Ding-Tao Wu, Yun-Xuan Zhao, Huan Guo, Ren-You Gan, Lian-Xin Peng, Gang Zhao, Liang Zou

**Affiliations:** 1 Key Laboratory of Coarse Cereal Processing, Ministry of Agriculture and Rural Affairs, Sichuan Engineering & Technology Research Center of Coarse Cereal Industralization, School of Food and Biological Engineering, Chengdu University, Chengdu 610106, China; ganrenyou@caas.cn (R.-Y.G.); penglianxin@cdu.edu.cn (L.-X.P.); zhaogang@cdu.edu.cn (G.Z.); 2 Institute of Food Processing and Safety, College of Food Science, Sichuan Agricultural University, Ya’an 625014, China; zhaoyunxuan0320@163.com (Y.-X.Z.); ghscny@163.com (H.G.); 3 Research Center for Plants and Human Health, Institute of Urban Agriculture, Chinese Academy of Agricultural Sciences, Chengdu 610213, China

**Keywords:** *Dictyophora indusiata* polysaccharide, extraction technique, structural properties, antioxidant activity, binding properties

## Abstract

In this study, different extraction techniques, including traditional hot water extraction (HWE), microwave-assisted extraction (MAE), pressurized assisted extraction (PAE), and ultrasonic-assisted extraction (UAE), were used to extract *Dictyophora indusiata* polysaccharides (DFPs), and their physicochemical and biological properties were compared. Results revealed that extraction yields of *D. indusiata* polysaccharides prepared by different extraction techniques ranged from 5.62% to 6.48%. *D. indusiata* polysaccharides prepared by different extraction techniques possessed similar chemical compositions and monosaccharide compositions, while exhibited different molecular weights (*M_w_*), apparent viscosities, and molar ratios of constituent monosaccharides. In particularly, *D. indusiata* polysaccharides prepared by HWE (DFP-H) had the highest *M_w_* and apparent viscosity among all DFPs, while *D. indusiata* polysaccharides extracted by UAE (DFP-U) possessed the lowest *M_w_* and apparent viscosity. In addition, the in vitro antioxidant effects of *D. indusiata* polysaccharides prepared by PAE (DFP-P) and DFP-U were significantly higher than that of others. Indeed, both DFP-P and DFP-H exhibited much higher in vitro binding properties, including fat, cholesterol, and bile acid binding properties, and lipase inhibitory effects than that of *D. indusiata* polysaccharides prepared by MAE (DFP-M) and DFP-U. These findings suggest that the PAE technique has good potential for the preparation of *D. indusiata* polysaccharides with desirable bioactivities for the application in the functional food industry.

## 1. Introduction

Polysaccharides are considered to be the major bioactive components in edible and medicinal mushrooms, which possess diverse health-promoting effects, such as anti-oxidant, anti-tumor, anti-inflammatory, immunomodulatory, anti-diabetic, and gut microbiota regulation effects [1,2,3]. Due to their diverse health-promoting effects, mushroom polysaccharides have been widely utilized for the prevention and treatment of diseases. In recent years, mushroom polysaccharides have also been extensively used in the food industry, such as functional foods, dietary supplements, and edible films for food packaging [1]. For example, *Inonotus obliquus* polysaccharides are developed as functional and health products for reducing blood fat, removing free radicals, and regulating immune function [4]. *Lentinus edodes* polysaccharides are incorporated in various food/beverage products to obtain fortified functional foods/beverages for enhancing immune function, lowering serum lipid, lowering blood glucose, and regulating gut microbiota [5]. In addition, polysaccharides extracted from *Ganoderma lucidum*, *Grifola frondosa*, and *Trametes versicolor* have been developed as dietary supplements for promoting immune function [1]. At present, the development of mushroom polysaccharides as functional foods has attracted increasing attention in the food industry.

As one of the most popular edible mushrooms in Asian countries, especially in China, *Dictyophora indusiata* is a saprophytic fungus belonging to the family *Phallaceae* [6]. It is also regarded as the “queen of mushrooms” because of its pleasing taste and appearance. It is demonstrated that *D*. *indusiata* possesses excellent nutritional and medicinal functions [7], such as anti-hyperlipidemic [8], anti-tumor [9], anti-inflammatory [7], neuroprotective [10], and hepatic-protective effects [11]. Polysaccharides, the major bioactive constituent of *D. indusiata*, have been plentifully studied due to their low biotoxicities and beneficial effects. Generally, β-1,3-glucan and galactan are the major bioactive polysaccharides in *D*. *indusiata* [6], which have attracted increasing attention in the functional food industry due to their various beneficial effects.

Different extraction techniques can lead to differences in the biological functions of natural polysaccharides [12]. Generally, traditional hot water extraction (HWE) is commonly used as a simple way to prepare polysaccharides, yet the disadvantages of HWE should also be considered, including comparably long operation time, comparably low extraction yields, and high operation temperature [13]. Therefore, several efficient extraction techniques, including microwave-assisted extraction (MAE), ultrasound-assisted extraction (UAE), and pressurized assisted extraction (PAE), have been introduced. Compared with HWE, MAE shows many advantages. It costs less time and solvents, and provides a high extraction yield [12,14]. UAE is also an emerging approach with many advantages, such as shortening extraction duration and economizing power consumption [13,15]. PAE, another extraction method, can increase the solubility of polysaccharides. PAE can also help infiltrate the solvent into the sample by reducing the surface tension and viscosity [16]. Indeed, although the structural features and biological functions of *D. indusiata* polysaccharides (DFPs) prepared by traditional hot water extraction [17,18], acid extraction [8], and enzyme-assisted extraction [19] have been reported, the physicochemical properties and biological functions of *D*. *indusiata* polysaccharides prepared by different efficient extraction techniques such as MAE, UAE, and PAE have seldom been compared. Therefore, it is worthwhile to investigate physicochemical and biological properties of DFPs prepared by different extraction techniques, which is beneficial for the improvement of their applications in the functional food industry.

This study aimed to investigate and compare physicochemical properties, in vitro antioxidant activities, and in vitro hypolipidemic activities (in vitro binding properties and inhibitory effects on lipase) of DFPs prepared by HWE, MAE, UAE, and PAE. Results from this work can provide practical and scientific foundations to choose satisfying extraction techniques to produce DFPs with desirable properties for application in the functional food industry.

## 2. Materials and Methods

### 2.1. Samples and Chemicals

The fruiting body of *D. indusiata* was bought from the local supermarket in Ya’an, China. The sample was dried, grounded, and sieved through a 60-mesh screen. The powders were stored at −20 °C before use. Carboxymethyl cellulose (CM-cellulose), cholesterol, sodium deoxycholate, sodium taurocholate, sodium glycocholate, sodium cholate, oleic acid, 1-phenyl-3-methyl-5-pyrazolone (PMP), monosaccharide standards, Griess reagent, 2,2-diphenyl-1-(2,4,6-trinitrophenyl) hydrazyl (DPPH), and 2,2′-azino-bis(3-ethylbenzothiazoline-6-sulphonic acid) (ABTS) were obtained from Sigma-Aldrich (Sigma, St. Louis, MO, USA). A free cholesterol assay kit was obtained from Solarbio (Solarbio, Beijing, China).

### 2.2. Preparation of D. Indusiata Polysaccharides (DFPs)

#### 2.2.1. Traditional Hot Water Extraction

**Figure 1:**
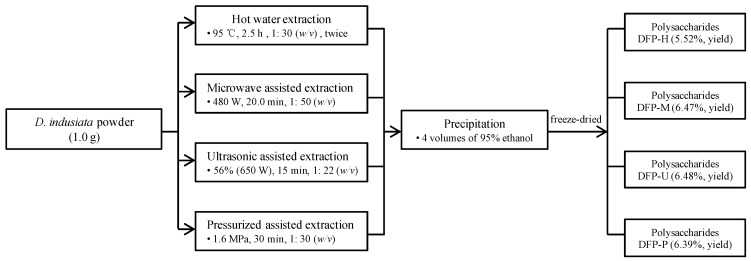
The flow chart for the extraction of polysaccharides from *D. indusiata* by different techniques. DFP-H, DFP-M, DFP-U, and DFP-P, *D. indusiata* polysaccharides prepared by hot water extraction, microwave assisted extraction, ultrasonic assisted extraction, and pressurized assisted extraction, respectively.

Traditional hot water extraction (HWE) was conducted as the method reported in our previous study with small adjustments [6]. Briefly, *D. indusiata* powders (1.0 g) were added into 35.0 mL of deionized water, and the polysaccharides were extracted twice at 95 °C (water bath) for 2.5 h. The water extract was obtained by centrifuging at 4000× *g* for 15 min, and four volumes of 95% ethanol (*v*/*v*) were then added and settled overnight. The precipitates were re-dissolved and freeze-dried, and coded as DPF-H. The phenol-sulfuric acid method and Bradford method were used to detect the total polysaccharides and proteins in DPF-H, respectively. A flow chart for the extraction of polysaccharides from *D. indusiata* was shown in Figure 1.

#### 2.2.2. Microwave Assisted Extraction

Microwave-assisted extraction (MAE) was also performed as the method reported in our previous study with minor modifications [20]. Briefly, *D. indusiata* powders (1.0 g) were added into deionized water (50.0 mL), and the polysaccharides were extracted with a microwave extractor (MKJ-J1-3, Makewave Company, Qingdao, China) for 20.0 min at 480 W and 85 °C. The water extract was obtained by centrifuging at 4000× *g* for 15 min, and four volumes of 95% ethanol (*v*/*v*) were added and settled overnight. The precipitates were re-dissolved and freeze-dried, and coded as DPF-M. Afterward, the phenol-sulfuric acid method and Bradford method were used to detect the total polysaccharides and proteins in DPF-M, respectively.

#### 2.2.3. Ultrasonic Assisted Extraction

Ultrasonic assisted extraction (UAE) was also conducted as our previous study [6]. Briefly, *D. indusiata* powders (1.0 g) were added into deionized water (22.0 mL), and the polysaccharides were extracted with an Ultrasonic Processor (650 W, 24 kHz, Scientz Company, Ningbo, China) for 15.0 min at room temperature, and ultrasonic amplitude was set at 56%. The water extract was obtained by centrifuging at 4000× *g* for 15 min, and four volumes of 95% ethanol (*v*/*v*) were added and settled overnight. The precipitates were re-dissolved and freeze-dried, and coded as DPF-U. Finally, the phenol-sulfuric acid method and Bradford method were used to detect the total polysaccharides and proteins in DPF-U, respectively.

#### 2.2.4. Pressurized Assisted Extraction

Pressurized assisted extraction (PAE) was also operated as our previous study with minor modifications [16]. Briefly, the *D*. *indusiata* powders (1.0 g) were added into deionized water (30.0 mL), and the polysaccharides were extracted with a laboratory-scale high-pressure reactor (LEC-300, Shanghai Laibei Scientific Instruments Co., Ltd., Shanghai, China) for 30.0 min at 1.6 MPa and 55 °C. The water extract was obtained by centrifuging at 4000× *g* for 15 min, and 4 volumes of 95% ethanol (*v*/*v*) were added and settled overnight. The precipitates were re-dissolved and freeze-dried, and coded as DPF-P. Finally, the phenol-sulfuric acid method and Bradford method were used to detect the total polysaccharides and proteins in DPF-P, respectively.

### 2.3. Physicochemical Characterization of DFPs

#### 2.3.1. Determination of Molecular Weights and Constituent Monosaccharides

The weight-average molecular weights (*M_w_*) of DFPs prepared by different extraction techniques were estimated by size exclusion chromatography coupled with a multi-angle laser light scattering and a refractive index detector (Wyatt Technology Co., Santa Barbara, CA, USA) [21]. A Shodex OHpak SB-806M HQ column (300 mm × 8.0 mm, i.d.) was used and set at 30 °C. The Astra software (version 7.1.3, Wyatt Technology Co., Santa Barbara, CA, USA) was utilized for data acquisition and analysis.

Constituent monosaccharides of DFPs were also evaluated by high-performance liquid chromatography followed by our previously established method [20]. Rha, Man, GlcA, GalA, Glc, Gal, Xyl, and Ara were used as standards. The constituent monosaccharides of DFPs were analyzed by an Agilent 1260 series LC system (Agilent Technologies, Palo Alto, CA, USA). The ZORBAX Eclipse XDB-C18 column (4.6 mm × 250 mm, i.d. 5 µm, Agilent Technologies Inc.) was used to separate monosaccharides. The mobile phase was 0.1 M phosphate buffer solution (pH = 6.7) and acetonitrile with a ratio of 83: 17 (*v*/*v*). The flow rate was set at 1.0 mL/min, and the wavelength of DAD was set at 245 nm.

#### 2.3.2. Determination of Apparent Viscosities

The apparent viscosities of DFPs prepared by different extraction techniques were measured by a Discovery Hybrid Rheometer-1 (DHR-1, TA Instruments, New Castle, DE, USA). It was equipped with a parallel steel plate (40 mm diameter, 1.0 mm gap) [22]. The concentration of each DFP was set at 2.5 mg/mL, and apparent viscosities were measured at 0.1 to 100 s^−1^. The temperature was set at 25 °C.

#### 2.3.3. Fourier Transform Infrared (FT-IR) Spectroscopy Analysis

The FT-IR spectroscopy analysis of DFPs prepared by different extraction techniques was conducted according to our previous study [20]. The Nicolet IS 10 FT-IR (Thermo Fisher Scientific, Waltham, MA, USA) was applied for the analysis of the FT-IR spectra of DFPs, which were recorded in the frequency range of 4000–400 cm^−1^.

### 2.4. Evaluation of In Vitro Antioxidant Activities and Hypolipidemic Activities of DFPs

#### 2.4.1. In Vitro Antioxidant Activities

The in vitro antioxidant activities of DFPs prepared by different extraction techniques, including DPPH, nitric oxide (NO), and ABTS radical scavenging activities, as well as reducing powers, were evaluated based on our previous study [20]. The NO and DPPH radical scavenging activities were determined at 1.0, 2.0, 3.0, 4.0, and 5.0 mg/mL, and the BHT (0.20–1.00 mg/mL) was utilized as positive control. The ABTS radical scavenging activities and reducing power were also determined at 1.0, 2.0, 3.0, 4.0, and 5.0 mg/mL, and the vitamin C (*V_c_*) was used as positive control. The ABTS radical scavenging activities and reducing power of *V_c_* were determined in the concentration range of 0.04–0.12 mg/mL and 0.10–0.30 mg/mL, respectively.

#### 2.4.2. In Vitro Hypolipidemic Activities

The in vitro binding properties (fat, cholesterol, and bile acid-binding properties) and inhibitory effects against the pancreatic lipase of DFPs prepared by different extraction techniques were measured based on our previous study [21]. The fat binding capacity of DFPs was calculated by the weight of bounded fat per weight of DFPs (g/g), and CM-cellulose was used as positive control. The cholesterol-binding activity of DFPs was calculated by milligram of binding cholesterol per gram of DPFs (mg/g), and CM-cellulose was used as positive control. The bile acid-binding activity of DFPs was expressed as percent of blank control (%), and cholestyramine was used as the positive control. The pancreatic lipase inhibitory effects of DFPs prepared by different extraction techniques were determined at 1.0, 2.0, 3.0, 4.0, and 5.0 mg/mL, and the orlistat was used as the positive control.

### 2.5. Statistical Analysis

Each experiment was performed three times, and the final data were expressed as means ± standard deviations. OriginLab 9.0 (OriginLab Corporation, Northampton, MA, USA) was applied for data analysis. Statistical differences (*p* < 0.05) were compared by one-way analysis of variance (ANOVA) with Duncan’s multiple range test.

## 3. Results and Discussion

### 3.1. Comparison of Physicochemical Characteristics of DFPs Prepared by Different Techniques

#### 3.1.1. Chemical Compositions of DFPs

Table 1 summarizes the basically chemical compositions and extraction yields of DFPs prepared by HWE, MAE, UAE, and PAE. It can be seen from Table 1 that the extraction yields of DFPs prepared by HWE, MAE, UAE, and PAE ranged from 5.62% to 6.48%. The extraction yields of DFPs were similar to the result of a previous study using ultrasound assisted extraction [6], but lower than that of an optimized hot water extraction [18] and an optimized enzyme-assisted extraction [19], which might be attributed to different resources of *D. indusiata* used. The extraction yields of DFP-M, DFP-U, and DFP-P were similar, which were higher than that of DFP-H. In addition, the total polysaccharides in DFPs were also influenced by different extraction techniques, similar to previously reported [16]. The total polysaccharides in DFP-H, DFP-M, DFP-U, and DFP-P were measured to be 83.68 ± 0.28%, 81.19 ± 0.33%, 80.37 ± 0.29%, and 86.17 ± 0.38%, respectively, indicating that polysaccharides were the major components in DFPs. A small amount of proteins were determined in DFPs, which ranged from 1.27% to 3.43%. The contents of total uronic acids in DFPs ranged from 1.94% to 3.16%. Compared with the HWE, UAE, MAE, and PAE not only saved extraction time, but also led to high extraction yields. In addition, the DFP-P obtained by the PAE method showed the highest content of total polysaccharides among all DFPs. Generally, pressurized assisted extraction can increase the solubility of polysaccharides, and contribute to the solvent infiltrate into the sample by reducing the surface tension and viscosity, thereby increasing the yields of polysaccharides [16,22]. The PAE method is worthy of application for the extraction of polysaccharides from *D*. *indusiata* in the food industry.

#### 3.1.2. Constituent Monosaccharides, Molecular Weights, and Apparent Viscosities of DFPs

**Figure 2:**
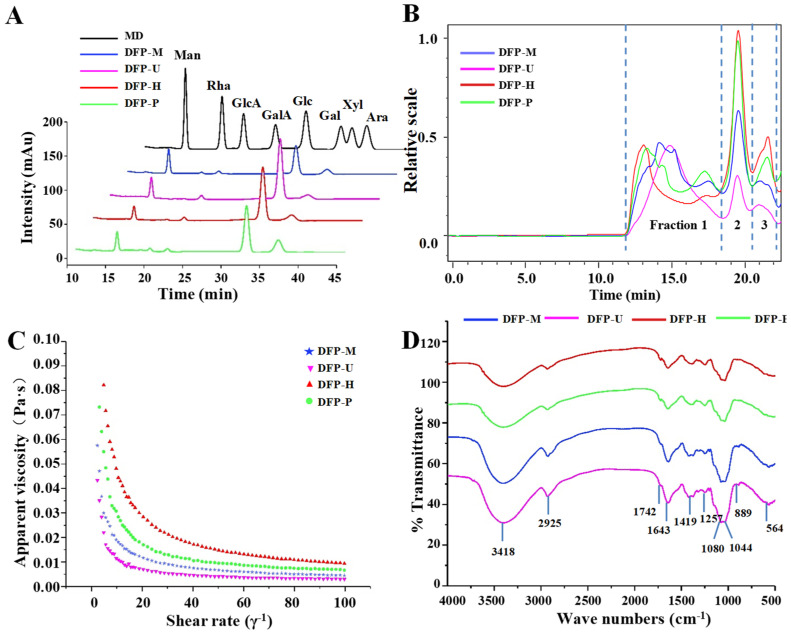
HPLC profiles (**A**); HPSEC chromatograms (**B**); apparent viscosities (**C**) and FT-IR spectra (**D**) of DFP-H, DFP-M, DFP-U, and DFP-P (*D. indusiata* polysaccharides prepared by HWE, MAE, UAE, and PAE, respectively); MD, mixed standard of monosaccharides; Man, mannose; Rha, rhamnose; GlcA, glucuronic acid; GalA, galacturonic acid; Glc, glucose; Gal, galactose; Xyl, xylose; Ara, arabinose. mAu, the signal intensity of monosaccharides derivatized with PMP detected by diode array detector.

The monosaccharides, molecular weights, and apparent viscosities are generally to be highly related to the biological activities of natural polysaccharides [23,24,25]. Thus, the constituent monosaccharides, molecular weights, and apparent viscosities of DFPs prepared by HWE, MAE, UAE, and PAE were measured. Figure 2A reveals that the HPLC-UV chromatograms of all DFPs prepared by different extraction techniques were similar. Previous results also indicated that compositional monosaccharides of DFP-H, DFP-M, DFP-U, and DFP-P were Man, Rha, GlcA, Gal, and Glc [11].

The molar ratios of the detected monosaccharides in DFP-H, DFP-M, DFP-U, and DFP-P are shown in Table 1, and the dominant monosaccharide in DFPs was Glc. The types of constituent monosaccharides in DFPs were not influenced by different methods, but their molar ratios were significantly influenced. The differences in constituent monosaccharides of natural polysaccharides obtained from different extraction methods were also reported by other studies [14,16].

Furthermore, Figure 2B displays the HPSEC-RID chromatograms of DFPs prepared by different extraction techniques, and three polysaccharide fractions (1 to 3) were detected. Fraction 1 was determined as the major polysaccharide fraction in DFPs, which obviously degraded during PAE, MAE, and UAE extraction (Figure 2B). Table 1 also shows the molecular weights of polysaccharide fractions (1 to 3) in *D*. *indusiata*. The molecular weights of polysaccharide fractions 1 to 3 in DFP-H, DFP-M, DFP-U, and DFP-P are also presented in Table 1, and varied from 108.97 × 10^4^ Da to 154.73 × 10^4^ Da (fraction 1), 9.82 × 10^4^ Da to 13.28 × 10^4^ Da (fraction 2), and 5.91 × 10^4^ Da to 7.80 × 10^4^ Da (fraction 3), respectively.

The order of molecular weights of polysaccharide fraction 1 in DFPs was DFP-H > DFP-P > DFP-M > DFP-U, suggesting that different extraction techniques remarkably influenced molecular weights of DFPs. Compared with the molecular weights of DFP-H, molecular weights of DFP-P were slightly lower, and DFP-M as well as DFP-U were significantly lower. Results showed that DFPs might degrade during the UAE, MAE, and PAE extraction, respectively. This phenomenon was similar to other studies that molecular weights of natural polysaccharides prepared by MAE and UAE were lower than that of HWE [15,16,20]. Furthermore, the polydispersities of fractions 1 to 3 in DFP-H, DFP-M, DFP-U, and DFP-P ranged from 1.64 to 2.01, from 1.07 to 1.28, and from 1.02 to 1.18, respectively, which were consistent with their HPSEC chromatograms.

Furthermore, the effect of shear rate on apparent viscosities of DFPs prepared by different techniques is shown in Figure 2C. The apparent viscosities of DFP-H, DFP-M, DFP-U, and DFP-P decreased with the increase of the shear rate (0.1–100 s^−1^). The order of apparent viscosities of DFPs was DFP-H > DFP-P > DFP-M > DFP-U. The apparent viscosities of DFPs were remarkably influenced by extraction techniques. Similar results were reported in other polysaccharides [16,25,26]. Indeed, PAE, MAE, and UAE significantly reduced the apparent viscosities of DFPs. This phenomenon was possibly caused by the decrease of their molecular weights and changes of their monosaccharide compositions. The findings suggest that the apparent viscosities of DFP-H, DFP-M, DFP-U, and DFP-P are highly associated with their molecular weights, which is also reported by other studies [27,28].

#### 3.1.3. FT-IR Spectra of DFPs

The structural characteristics of DFPs were measured by FT-IR. Figure 2D shows the FT-IR spectra of DFP-H, DFP-M, DFP-U, and DFP-P. DFPs prepared by different extraction techniques showed similar FT-IR spectra, that displayed similar absorption peaks. The broad peaks at 3418 cm^−1^ and 2925 cm^−1^ are influenced by the hydroxyl group stretching vibration and C-H asymmetric stretching vibration [8,20]. The absorption band at 1742 cm^−1^ is the C=O stretching vibration of esterified groups, indicating the presence of uronic acids [15]. The absorption peak at 1643 cm^−1^ is attributed to the stretching vibration of C=O, and the absorption peak at 1419 cm^−1^ is assigned to the C–H variable angle vibration [15]. The absorptions in the region of 1000–1200 cm^−1^ due to stretching vibrations of C–OH side groups and the C–O–C glycosidic band [11]. The absorption peak at 889 cm^−1^ is the typical absorption peak of the C-H in β-linkage, indicating that DFPs contains β-glucan. The findings were similar to the results of Deng et al. [17]. The results suggested that the types of glycosidic bonds and configurations of polysaccharides extracted from *D*. *indusiata* were not changed by different extraction techniques, similar to previously reported [15].

### 3.2. Comparison of In Vitro Antioxidant Activities of DFPs Prepared by Different Extraction Techniques

**Figure 3:**
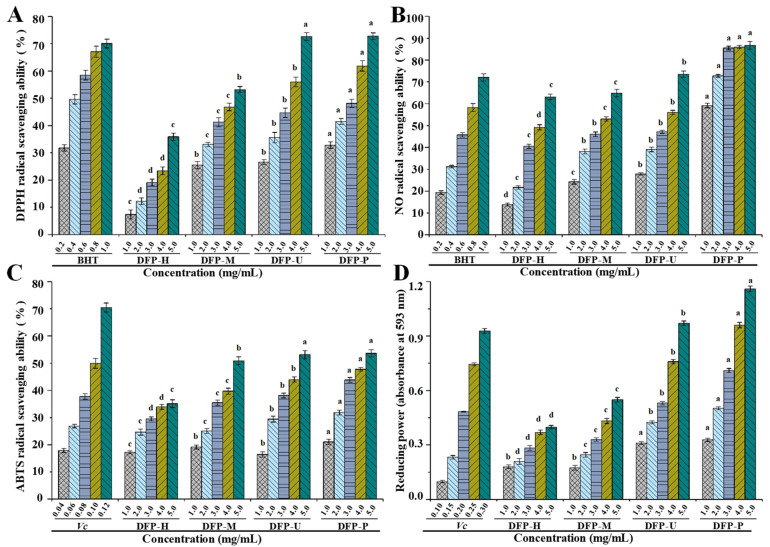
DPPH (**A**); NO (**B**) and ABTS (**C**) radical scavenging activities and reducing powers (**D**) of DFP-H, DFP-M, DFP-U, and DFP-P (*D. indusiata* polysaccharides prepared by HWE, MAE, UAE, and PAE, respectively); The error bars are standard deviations; Significant (*p* < 0.05) differences among in vitro antioxidant activities of *D. indusiata* polysaccharides prepared by different extraction methods at the same concentration are shown by data bearing different letters (a–d), respectively; Statistical significances were carried out by ANOVA plus *post hoc* Duncan’s test.

The free radical scavenging activity of antioxidants is commonly assessed by the DPPH radical scavenging method [29], therefore, the DPPH radical scavenging capabilities of DFPs extracted by the four methods were measured and compared. The DPPH radical scavenging activities of DFP-H, DFP-M, DFP-U, and DFP-P are shown in Figure 3A, where DFP-H, DFP-M, DFP-U, and DFP-P show noticeable DPPH radical scavenging capabilities. 

At the concentration of 5.0 mg/mL, the DPPH radical scavenging activities of DFP-H, DFP-M, DFP-U, and DFP-P were measured to be 35.88 ± 1.31%, 53.18 ± 1.12%, 72.59 ± 1.40%, and 72.77 ± 1.26%, respectively. In addition, the IC_50_ value of DPPH radical scavenging activity of DFP-P was determined to be 2.473 mg/mL. Compared with the BHT (IC_50_ = 0.418 mg/mL), DFP-P also exerted relatively good DPPH radical scavenging activity. Different extraction methods exhibited obvious influences on the DPPH radical scavenging activities of DFPs, which were similar to other studies [16,29,30]. However, Deng et al. [31] found that the DPPH radical scavenging activity of polysaccharides extracted from *D*. *indusiata* was about 50% at 1.0 mg/mL. This result was higher than that of DFP-H, DFP-P, DFP-M, and DFP-U. In particular, the DPPH radical scavenging activities of DFP-U and DFP-P were similar, which were higher than those of DFP-M and DFP-H.

Additionally, as shown in Figure 3B, DFPs also exerted remarkable nitric oxide (NO) radical scavenging activities in a dose-dependent manner. At the concentration of 5.0 mg/mL, the NO radical scavenging activities of DFP-H, DFP-M, DFP-U, and DFP-P were measured to be 63.14 ± 1.30%, 64.91 ± 1.76%, 73.51 ± 1.43%, and 86.69 ± 1.80%, respectively. In addition, the IC_50_ value of NO radical scavenging activity of DFP-P was determined to be 0.698 mg/mL. Compared with the *V_c_* (IC_50_ = 0.662 mg/mL), DFP-P also exerted strong NO radical scavenging activity. Different extraction techniques also significantly influenced the NO radical scavenging activities of DFPs, and DFP-P extracted by the PAE technique exhibited obviously stronger NO radical scavenging activities than that of others.

ABTS radical is also commonly applied for the determination of the total antioxidant capacity of various compounds [29]. The ABTS radical scavenging activities of DFP-H, DFP-M, DFP-U, and DFP-P are shown in Figure 3C. Likewise, DFPs prepared by different extraction techniques exhibited obvious ABTS radical scavenging activities with dose-dependent manners. At the concentration of 5.0 mg/mL, the ABTS radical scavenging activities of DFP-H, DFP-M, DFP-U, and DFP-P were measured to be 35.11 ± 1.43%, 50.90 ± 1.45%, 53.12 ± 1.54%, and 53.62 ± 1.34%, respectively. Additionally, the ABTS radical scavenging activity of DFP-P (IC_50_ = 4.266 mg/mL) was lower than that of *V_c_* (IC_50_ = 0.104 mg/mL). Results showed that ABTS radical scavenging activities of DFP-U and DFP-P were similar, which were higher than that of DFP-M and DFP-H.

For the measurement of the reducing capability, the Fe^3+^–Fe^2+^ transformation in the presence of DFPs prepared by different extraction techniques was investigated by the potassium ferricyanide reduction method [17]. The reducing powers of DFP-H, DFP-M, DFP-U, and DFP-P are shown in Figure 3D. It is seen from Figure 3D that DFP-P also showed the highest reducing power among all samples at the concentrations ranged from 1.0 to 5.0 mg/mL (*p* < 0.05), followed by DFP-U and DFP-M, and DFP-H displayed the lowest reducing power. At the concentration of 5.0 mg/mL, the absorbances of reducing powers of DFP-H, DFP-M, DFP-U, and DFP-P were measured to be 0.40 ± 0.02, 0.55 ± 0.02, 0.97 ± 0.03, and 1.16 ± 0.03, respectively. The reducing power of DFP-P was lower than that of *V_c_*. The findings indicated that the reducing powers of DFPs were obviously influenced by different extraction techniques, similar results can also be found in other studies [20,32,33]. Studies have shown that the antioxidant activities of natural polysaccharides are associated with their structure features, compositional monosaccharides, and molecular weights [23,24,25]. Simultaneously, the physicochemical characteristics of *D*. *indusiata* polysaccharides were influenced by different extraction techniques [34]. In the present study, the higher antioxidant activities observed in DFP-P and DFP-U might be partially attributed to their contents of total polysaccharides, constituent monosaccharides, and molecular weights [35]. Furthermore, it is worth noting that the presence of electrophilic groups like aldehyde or keto can promote the liberation of hydrogen from O-H bond, and these groups can also improve the antioxidant activities of natural polysaccharides [36].

### 3.3. Comparison of In Vitro Hypolipidemic Activities of DFPs Prepared by Different Extraction Techniques

#### 3.3.1. Binding Properties of DFPs

**Figure 4:**
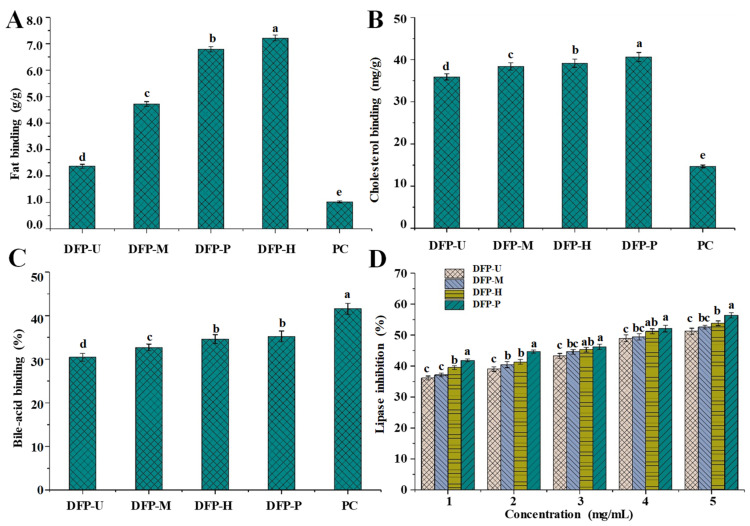
The in vitro fat-binding (**A**); cholesterol-binding (**B**); bile-acid binding (**C**) capacities, and in vitro inhibitory effects on the pancreatic lipase (**D**) of DFP-H, DFP-M, DFP-U, and DFP-P (*D. indusiata* polysaccharides extracted by HWE, MAE, UAE, and PAE respectively); PC, positive control; Values represent mean ± standard deviation, and the letters (a–e) differ significantly (*p* < 0.05) among fat-binding, cholesterol-binding, bile-acid binding capacities, and inhibitory effects on the pancreatic lipase of *D. indusiata* polysaccharides prepared by different extraction methods and the positive control, respectively. Statistical significances were carried out by ANOVA plus *post hoc* Duncan’s test.

Numerous complications and hyperlipidemia are reported to be related to the excessive intake of fat and cholesterol, including atherosclerosis, cerebral infarction, cardiovascular diseases, diabetes, hemiplegia, stroke, cancer, and so on [8,37]. Carbohydrates, especially polysaccharides, exhibit obvious abilities to lower blood fat and cholesterol, which may contribute to their hypolipidemic activities [21,23]. Figure 4 shows the in vitro binding properties of DFPs prepared by different extraction techniques. The fat (Figure 4A), cholesterol (Figure 4B), and bile acid (Figure 4C) binding abilities of DFP-H, DFP-M, DFP-U, and DFP-P ranged from 2.37 ± 0.07 to 7.17 ± 0.10 g/g, from 35.91 ± 0.74 to 40.61 ± 1.09 mg/g, and from 30.45 ± 0.89% to 35.24 ± 1.26%, respectively.

Different extraction techniques led to a variation of in vitro binding capacity of DFPs, which was consistent with other studies [16]. Indeed, DFP-P and DFP-H had higher fat binding abilities than that of DFP-U and DFP-M, which were also higher than that of polysaccharides extracted from potatoes peels (4.398 ± 0.04 g/g) [38], but lower than that of polysaccharides extracted from *Lilium lancifolium* Thunb (9.25 ± 0.17 g/g) [39]. Similarly, DFP-P had the highest cholesterol-binding ability among all samples, which was 40.61 ± 1.09 mg/g. In addition, both DFP-P and DFP-H showed significantly higher bile acid-binding ability, respectively, followed by lower in DFP-M, and the lowest in DFP-U. The results further suggested that the PAE was an outstanding extraction technique because DFP-P had relatively high binding capacities. Several findings have demonstrated that the in vitro binding capacities of polysaccharides are correlated with their apparent viscosities and molecular weights [16,40], which may be due to the high hydrophobic interaction between the fat/cholesterol and polysaccharides [41]. The high molecular weight can reduce the water solubility of DFP-H and DFP-P, resulting in the decrease of their hydrophilicity, which can increase their affinity for the fat/cholesterol by increasing the aggregation on their surface [41]. Due to the solubility in the aqueous condition, *D.* *indusiata* polysaccharides may have a similar binding mechanism to that of water-soluble fibers. The binding effects of DFP-H and DFP-P were also primarily correlated to their high apparent viscosities. In conclusion, the relatively high binding capacities of DFP-P and DFP-H might be caused by their high molecular weights and apparent viscosities. Moreover, compared with the positive controls, DFPs prepared by different techniques exerted better fat and cholesterol binding abilities. Although the bile acid-binding ability of DFPs was lower than that of the positive control, DFPs still showed apparent bile acid-binding capacities. These results confirmed the potential of DFPs used as functional foods/drugs recipes to prevent hyperlipidemia.

#### 3.3.2. In Vitro Inhibitory Effects of DFPs on the Pancreatic Lipase

Epidemiological studies have proved that obesity is a risk factor for certain fatal cancers, such as pancreatic ductal adenocarcinoma [42]. Hyperlipidemia is a major risk factor resulting in numerous complications including cardiovascular diseases, atherosclerosis, fatty liver, stroke, diabetes, cerebral infarction, myocardial infarction, hemiplegia, and so on [8,43]. Previous studies have shown that natural polysaccharides exhibit obvious inhibitory effects on the pancreatic lipase [21]. Thus, the present study evaluated the inhibitory effects of DFPs on the pancreatic lipase. The inhibitory activities of DFPs on the pancreatic lipase are summarized in Figure 4D. DFPs prepared by different extraction techniques were able to inhibit the activity of pancreatic lipase, and the inhibitory effects were dose-dependent. The inhibitory effects of DFPs on the pancreatic lipase varied with different extraction techniques. At the concentration of 5.0 mg/mL, the inhibitions on the pancreatic lipase of DFP-H, DFP-M, DFP-U, and DFP-P were measured to be 58.39 ± 0.98%, 52.65 ± 0.83%, 54.67 ± 1.03%, and 63.20 ± 1.11%, respectively. Previous studies have shown that natural polysaccharides are capable to limit fat digestion by inhibiting the pancreatic lipase activity, and regulate hyperlipidemia and obesity [44,45]. In addition, the inhibitory effects of DFPs on the pancreatic lipase were also positively correlated with their apparent viscosities and molecular weights. In this study, DFPs could inhibit lipase activity to a certain extent might be due to the binding of hydrophobic interaction and electrostatic interaction, resulting in a change in the conformation of lipase, or they could absorb and encapsulate lipase [46]. The highest inhibitory efficiency on the pancreatic lipase was shown by DFP-P, indicating PAE is an efficient method for extracting polysaccharides with relatively high bioactivities. At present, some synthetic drugs, such as statins, have been widely used to treat obesity and hyperlipidemia, but they usually have side effects and are not good for human health [8]. Therefore, there is an urgent need to find new and innovative ways to treat hyperlipidemia. In summary, DFPs, especially DFP-P extracted by PAE, had good potential to be used as functional foods for preventing hyperlipidemia.

## 4. Conclusions

In the present study, different efficient extraction techniques were applied to extract *D*. *indusiata* polysaccharides. The results revealed that extraction yields of DFP-M, DFP-U, and DFP-P were similar, which were higher than that of DFP-H. DFPs prepared by different extraction techniques had the same monosaccharide compositions but a slight difference in their molar ratios. In addition, DFP-H had the largest molecular weight and apparent viscosity among all samples, but its antioxidant activity was the weakest among all samples. The strong in vitro antioxidant activity, in vitro binding property, and inhibitory effect on pancreatic lipase were detected in DFP-P. Overall, these findings suggest that the PAE technique can be an efficient method for the preparation of *D. indusiata* polysaccharides with desirable bioactivities for application in the food industry.

## Figures and Tables

**Table 1:** Chemical compositions, molecular weights (*M_w_*), and compositional monosaccharides of DFP-H, DFP-M, DFP-U, and DFP-P.

Samples	DFP-H	DFP-M	DFP-P	DFP-U
**Extraction yield (%)**	5.52 ± 0.14 ^b^	6.47 ± 0.17 ^a^	6.39 ± 0.13 ^a^	6.48 ± 0.12 ^a^
**Total polysaccharides (%)**	83.68 ± 0.28 ^b^	81.19 ± 0.33 ^c^	86.17 ± 0.38 ^a^	80.37 ± 0.29 ^d^
**Proteins (%)**	2.07 ± 0.08 ^c^	3.43 ± 0.15 ^a^	2.84 ± 0.12 ^b^	1.27 ± 0.13 ^d^
**Total uronic acids (%)**	1.94 ± 0.07 ^c^	2.82 ± 0.09 ^b^	1.99 ± 0.09 ^c^	3.16 ± 0.11 ^a^
***M_w_* × 10^4^ (Da)**				
**Fraction 1**	154.73	132.02	147.29	108.97
**Fraction 2**	12.65	13.28	9.82	10.09
**Fraction 3**	6.68	7.80	5.91	6.11
***M_w_*/*M_n_***				
**Fraction 1**	1.94	2.01	1.96	1.64
**Fraction 2**	1.07	1.24	1.28	1.23
**Fraction 3**	1.18	1.02	1.05	1.02
**Monosaccharides and molar ratios**				
**Mannose**	2.68	4.19	3.30	2.68
**Rhamnose**	1.00	1.00	1.00	1.00
**Glucuronic acid**	0.98	0.44	0.75	0.46
**Galactose**	2.13	1.74	3.59	1.24
**Glucose**	10.50	10.70	7.91	8.83

DFP-H, DFP-M, DFP-U, and DFP-P are the *D. indusiata* polysaccharides prepared by HWE, MAE, UAE, and PAE, respectively; Values represent mean ± standard deviation, and superscripts a–d differ significantly (*p* < 0.05) among extraction yields, total polysaccharides, and total uronic acids of *D. indusiata* polysaccharides prepared by different extraction methods, respectively; Statistical significances were carried out by ANOVA plus post hoc Ducan’s test. Fractions 1–3 were the same in Figure 2B.

## Data Availability

Not applicable.

## Abbreviations

ABTS, 2,2′-azino-bis (3-ethylbenzothiazoline-6-sulphonic acid; Ara, arabinose; BHT, butylated hydroxytoluene; DPPH, 2,2-diphenyl-1-(2,4,6-trinitrophenyl) hydrazyl; DFPs, *Dictyophora indusiata* polysaccharides; DFP-H, *D**. indusiata* polysaccharides prepared by hot water extraction; DFP-U, *D**. indusiata* polysaccharides extracted by ultrasonic-assisted extraction; DFP-P, *D**. indusiata* polysaccharides prepared by pressurized assisted extraction; DFP-M, *D**. indusiata* polysaccharides prepared by microwave-assisted extraction; FT-IR, fourier transform infrared spectroscopy; GlcA, glucouronic acid; GalA, galactouronic acid; Glc, glucose; Gal, galactose; HWE, hot water extraction; MAE, microwave-assisted extraction; Man, mannose; *M_w_*, molecular weights; NO, nitric oxide; PAE, pressurized assisted extraction; PMP, 1-phenyl-3-methyl-5-pyrazolone; Rha, rhamnose; UAE, ultrasonic-assisted extraction; Xyl, xylose.
